# Phase Ib dose-finding study of axitinib plus pembrolizumab in treatment-naïve patients with advanced renal cell carcinoma

**DOI:** 10.1186/2051-1426-3-S2-P353

**Published:** 2015-11-04

**Authors:** Michael B Atkins, Shilpa Gupta, Toni K Choueiri, David F McDermott, Igor Puzanov, Jamal Tarazi, Stephen Keefe, Brad Rosbrook, Debasis Chakrabarti, Elizabeth R Plimack

**Affiliations:** 1 The Cytokine Working Group; Department of Medicine, Georgetown-Lombardi Comprehensive Cancer Center, Washington, DC, USA; 2 Masonic Cancer Center, University of Minnesota, Minneapolis, MN, USA; 3 Department of Medicine, Dana-Farber Cancer Institute, Boston, MA, USA; 4 The Cytokine Working Group; Division of Hematology/Oncology, Beth Israel Deaconess Medical Center, Boston, MA, USA; 5 Vanderbilt University Medical Center, Nashville, TN, USA; 6 Pfizer Oncology, San Diego, CA, USA; 7 Merck Research Laboratories, North Wales, PA, USA; 8 Pfizer Oncology, Collegeville, PA, USA; 9 Department of Medical Oncology, Fox Chase Cancer Center, Philadelphia, PA, USA

## Background

Axitinib, an inhibitor of vascular endothelial growth factor receptors (VEGFRs), is approved for second-line treatment of advanced renal cell carcinoma (aRCC). Pembrolizumab is a humanized monoclonal antibody that blocks binding of the immune-checkpoint receptor programmed death-1 (PD 1) to its ligands (PD-L1/2). Combination treatment with PD-1 and VEGFR inhibitors has shown benefit in aRCC, but increased toxicity has been observed. This ongoing Phase Ib study (NCT02133742) is assessing safety and tolerability of axitinib plus pembrolizumab in treatment-naïve patients with aRCC to estimate the maximum tolerated dose (MTD) and recommended Phase II dose (RP2D).

## Methods

Eligible patients have clear-cell aRCC, primary tumor resection, ≥1 measurable lesion, Eastern Cooperative Oncology Group performance status 0/1, no pre-existing uncontrolled hypertension, and no prior systemic therapy for aRCC; tumor biospecimens are mandatory. Axitinib is administered orally (starting dose: 5 mg twice daily) beginning on day –7; pembrolizumab is administered intravenously (2 mg/kg) on day 1 of each 3-week cycle. MTD was estimated by modified toxicity probability interval method. Tumors are assessed per Response Evaluation Criteria in Solid Tumors v1.1 at baseline, week 12, and every 6 weeks thereafter. Primary endpoint is dose-limiting toxicities (DLTs) during the first 2 cycles. Secondary endpoints include safety, tumor response, and biomarkers.

## Results

As of June 23, 2015, 11 patients (8 males; mean age 61 years) were enrolled; 1 was not evaluable for DLTs. Three DLTs were reported: transient ischemic attack (TIA; n=1) and < 75% of planned axitinib dose received due to treatment-related toxicity (n=2). MTD and RP2D were determined to be axitinib 5 mg twice daily plus pembrolizumab 2 mg/kg every 3 weeks. Ten patients remain on treatment (3 patients with DLTs were administered reduced axitinib doses) and progression-free. Five patients have had confirmed partial responses; 5 have stable disease with 4–33% tumor shrinkage. Common all-grade adverse events were diarrhea and hypothyroidism (n=6 each) and arthralgia, fatigue, and headache (n=4 each); 2 patients had increased hepatic transaminases (alanine aminotransferase and aspartate aminotransferase). Grade 3/4 adverse events were headache, increased alanine aminotransferase and aspartate aminotransferase, hyperuricemia, acute cholecystitis, and TIA (n=1 each). Only 1 of 9 evaluable tumor biospecimens was PD-L1+; this patient had stable disease.

## Conclusions

Preliminary results indicate axitinib plus pembrolizumab is well tolerated at standard doses of each agent and exhibits antitumor activity in treatment-naïve patients with aRCC. Additional patients are being enrolled to confirm the RP2D and further evaluate safety and activity of this combination.

